# Niemann–Pick C1-like 1 as a Prognostic Marker in Renal Cell Carcinoma: A Retrospective Cohort Study

**DOI:** 10.3390/life14111444

**Published:** 2024-11-07

**Authors:** Ryuk Jun Kwon, Ho Jun Kim, Young-Shin Lee, Hye Sun Lee, Sang Yeoup Lee, Eun-Ju Park, Youngin Lee, Sae Rom Lee, Jung-In Choi, Soo Min Son, Jeong Gyu Lee, Yu Hyeon Yi, Young Jin Tak, Seung-Hun Lee, Gyu Lee Kim, Young Jin Ra, Young Hye Cho

**Affiliations:** 1 Family Medicine Clinic and Research Institute for Convergence of Biomedical Science and Technology, Pusan National University Yangsan Hospital, Yangsan 50612, Republic of Korea; brain6@hanmail.net (R.J.K.);; 2 Department of Family Medicine, Pusan National University School of Medicine, Yangsan 50612, Republic of Koreagreatseunghun@daum.net (S.-H.L.);; 3 Graduate School of Health Science and Technology, College of Information and Biotechnology, Ulsan National Institute of Science and Technology, Ulsan 44919, Republic of Korea; 4 Department of Medical Education, Pusan National University School of Medicine, Yangsan 50612, Republic of Korea; 5 Department of Family Medicine and Biomedical Research Institute, Pusan National University Hospital, Busan 49241, Republic of Korea

**Keywords:** cholesterol, ezetimibe, lipid metabolism, NPC1L1, prognostic marker, renal cell carcinoma

## Abstract

Background: Renal cell carcinoma (RCC) is a highly aggressive malignancy accounting for the majority of kidney cancers. Despite recent advancements in therapeutic options, the prognosis for advanced-stage RCC remains poor. Niemann–Pick C1-Like 1 (NPC1L1) plays a crucial role in cholesterol absorption and has been implicated in cancer progression across various cancers. However, its expression patterns and prognostic significance in RCC remain unclear. Methods: In this study, NPC1L1 expression in normal and RCC tissues, including subtypes, was compared using TCGA, GEPIA2, and The Human Protein Atlas. Clinical correlations were assessed, and the impact of *NPC1L1* on overall survival (OS) and progression-free survival (PFS) was evaluated. Gene effect scores were analyzed using the DepMap tool to determine the involvement of *NPC1L1* in RCC progression. Results: NPC1L1 expression was significantly lower in RCC tissues compared to normal tissues, particularly in the clear cell RCC (ccRCC), papillary RCC (pRCC), and chromophobe RCC (chRCC) subtypes, but increased in advanced tumor stages. Higher *NPC1L1* expression was associated with worse OS and PFS in RCC patients. Multivariable Cox regression confirmed *NPC1L1* as an independent prognostic marker. Additionally, gene effect scores showed that NPC1L1 is essential for the survival of specific RCC cell lines. Conclusions: This study determines NPC1L1 as an independent prognostic indicator in RCC, with higher expression associated with poor survival outcomes. These findings suggest that NPC1L1 could serve as a valuable marker for identifying high-risk RCC patients. Further research is required to investigate the molecular mechanisms underlying the role of NPC1L1 in RCC progression.

## 1. Introduction

Renal cell carcinoma (RCC) constitutes the majority of kidney cancers and is often aggressive, especially in its advanced stages [1]. Its risk factors include hypertension, smoking, obesity, acquired cystic kidney disease, exposure to trichloroethylene, and genetic syndromes like von Hippel–Lindau disease [2]. The incidence of RCC has steadily risen, with over 76,000 new cases diagnosed annually in the United States alone [3]. Metastatic RCC has a significant mortality rate, with median overall survival (OS) ranging between 23 and 42 months, depending on the treatment approach such as immunotherapy or targeted therapies [4]. Treatment for RCC typically involves nephrectomy for localized tumors, while advanced cases are managed with immune checkpoint inhibitors and VEGF-targeted therapies [5]. New molecular therapies targeting pathways involving HIF2α and mTOR also show promise, particularly for clear cell RCC [6]. Despite these advancements in therapeutic options, the prognosis for patients with advanced-stage RCC remains poor, highlighting the need for reliable prognostic markers to guide clinical decisions and improve patient outcomes.

RCC subtypes, including clear cell RCC (ccRCC), papillary RCC (pRCC), and chromophobe RCC (chRCC), are classified based on histological features and primarily originate from tubular epithelial cells [7]. These subtypes exhibit significant metabolic alterations within the renal tubules, contributing to the development and progression of RCC [8]. For example, in RCC, especially ccRCC, significant changes in lipid metabolism occur, with enhanced lipid accumulation and alterations in fatty acid and cholesterol pathways that contribute to tumor progression [9]. Cholesterol metabolism modulates cancer hallmarks, such as autophagy and immune evasion, to facilitate tumor growth and metastasis in RCC and other cancers [10]. Cholesterol dysregulation enhances metastatic capacity by promoting resistance to ferroptosis, a form of cell death critical for preventing cancer spread [11]. Additionally, cholesterol accumulation within ccRCC cells has been shown to drive migration and invasion via the KLF5/miR-27a/FBXW7 pathway [12]. Among various molecules associated with cholesterol metabolism, Niemann–Pick C1-Like 1 (NPC1L1) is a key transmembrane protein in cholesterol absorption [13]. It is the pharmacological target of ezetimibe, a drug used to treat uncontrolled dyslipidemia [14]. High levels of NPC1L1 are observed in the human small intestine and liver, but its presence is minimal in the colon, kidneys, and brain [15]. Recent studies have suggested that NPC1L1 may play a role in cancer progression, with variable expression levels reported in different cancer types. For example, NPC1L1 expression is downregulated in hepatocellular carcinoma but upregulated in pancreatic and colon cancers [15,16,17]. However, its expression patterns and potential prognostic significance in RCC remain largely unexplored.

In the present study, TCGA data were used to compare *NPC1L1* expression between normal tissues and RCC, including its subtypes, and further analyzed these differences using the GEPIA2 and The Human Protein Atlas platforms. Moreover, correlations between *NPC1L1* expression and clinical characteristics were investigated, while its influence on OS and progression-free survival (PFS) in RCC patients was examined, determining its potential as an independent prognostic factor for RCC. To assess the gene effect scores of NPC1L1 in RCC, the DepMap tool from the UALCAN website was used.

## 2. Materials and Methods

### 2.1. Patients and Clinical Information

Clinical datasets for RCC patients were sourced from The Cancer Genome Atlas (TCGA). TCGA is a large-scale project initiated by the National Cancer Institute (NCI) and National Human Genome Research Institute (NHGRI) to systematically characterize the genomic and molecular alterations across 33 cancer types, including RCC. TCGA provides publicly accessible data derived from patient tumor and matched normal tissue samples, encompassing DNA sequencing, RNA sequencing, copy number variation (CNV), and DNA methylation analyses. Available through the Genomic Data Commons (GDC) portal, these data enable researchers to explore molecular signatures, genomic alterations, and expression patterns in various cancers. For this study, patient datasets were accessed through the cBioPortal platform (http://www.cbioportal.org/), which provides an organized version of TCGA cancer data (accessed on 1 March 2020). Initially, 860 RCC patients were identified, but after excluding 32 patients without gene expression or clinical data, 828 patients remained for analysis. This cohort included 510 patients with clear cell RCC, 253 with papillary RCC, and 65 with chromophobe RCC (Figure S1). To compare NPC1L1 expression between normal and RCC tissues, data from the GDAC platform (http://gdac.broadinstitute.org/) was used (accessed on 1 March 2020). 

Relevant clinical characteristics such as patient age, sex, histological subtype, tumor stage (T), nodal involvement (N), metastasis (M), American Joint Committee on Cancer (AJCC) stage, OS, and PFS were included in the analysis. Histological subtypes were categorized into clear cell, papillary, and chromophobe RCC. OS was defined as the time from diagnosis to death from any cause or last follow-up, while PFS was defined as the period from treatment initiation to the first occurrence of cancer progression or relapse. 

### 2.2. Analysis of Gene Expression and Gene Effect Scores Using Online Platforms

The GEPIA2 online platform (http://gepia2.cancer-pku.cn/) was used to compare the median expression levels of *NPC1L1* between normal and tumor tissues across multiple organs, with a focus on kidney tissues (accessed on 2 September 2024). Gene expression is displayed using TPM (Transcripts Per Million), which is defined as a normalization method for RNA-Seq data that scales read counts by transcript length and adjusts all values to a per-million scale. The histological analysis of NPC1L1 protein expression was conducted using data from The Human Protein Atlas (http://www.proteinatlas.org/) (accessed on 2 September 2024). Tissue samples, including both normal and tumor tissues, were stained using the CAB070127 antibody to detect NPC1L1 protein expression. To assess the gene effect scores of NPC1L1 in RCC, the DepMap tool provided by the UALCAN website (http://ualcan.path.uab.edu) was utilized (accessed on 5 September 2024). The gene effect scores for NPC1L1 were retrieved from the Cancer Dependency Map (DepMap) database, which offers CRISPR-based gene knockout screening data. This tool enables the identification of gene dependencies in RCC cell lines. The gene effect scores were analyzed to determine the involvement of NPC1L1 in RCC progression and survival.

### 2.3. Statistical Analysis

Descriptive statistics were used to analyze the histological types, gender, and staging of patients with RCC. Patient age and overall survival rates were presented as mean ± standard deviation. RSEM (RNA-Seq by Expectation-Maximization) was used to estimate *NPC1L1* gene expression levels by normalizing for sequencing depth and gene length, allowing for reliable comparisons across samples. Expression values of 0 were excluded for log2-transformation of RSEM values. T-tests were then performed using these RSEM values to compare *NPC1L1* expression between normal tissues and RCC, between normal tissues and RCC subtypes, and between low-stage (I, II) and high-stage (III, IV) tumors. A chi-square test was conducted to assess the association between *NPC1L1* expression and patient characteristics. For Kaplan–Meier analysis, RCC patients were divided into low and high *NPC1L1* expression groups based on the median expression value. Since the median survival period could not be calculated for certain groups due to low event rates, the mean survival period and the 75th percentile survival period were used. To determine whether *NPC1L1* is an independent prognostic factor for survival, both univariable and multivariable Cox proportional hazard models were applied. Statistical analyses were conducted using SPSS 20, with significance set at *p* < 0.05.

## 3. Results

### 3.1. RCC Patient Characteristics

The general characteristics of patients with RCC were analyzed (Table 1). Of the 828 patients, 553 (66.8%) were men and 275 (33.2%) were women. The proportion of RCC subtypes were 61.6% for clear cell RCC, 30.6% for papillary RCC, and 7.8% for chromophobe RCC. The average age of all patients was 60.11 ± 12.52 years and the mean OS was 42.97 ± 33.66 months. For the T stages, 560 patients (67.6%) were classified as T1–T2, while 266 patients (32.1%) were classified as T3–T4. As for the M stages, M0 represented 518 patients (62.6%), and M1 represented 89 patients (10.7%). In terms of the N stages, N0 included 313 individuals (37.8%), and N1–N2 encompassed 48 individuals (5.8%). In AJCC stage, Stage I, Stage II, Stage III, and Stage IV were 437 (52.8%), 100 (12.0%), 187 (22.6%), and 104 patients (12.6%), respectively. In addition, the general characteristics of each subtype are also shown in Table 1.

### 3.2. NPC1L1 Expression in Normal and RCC Tissues

**Figure 1:**
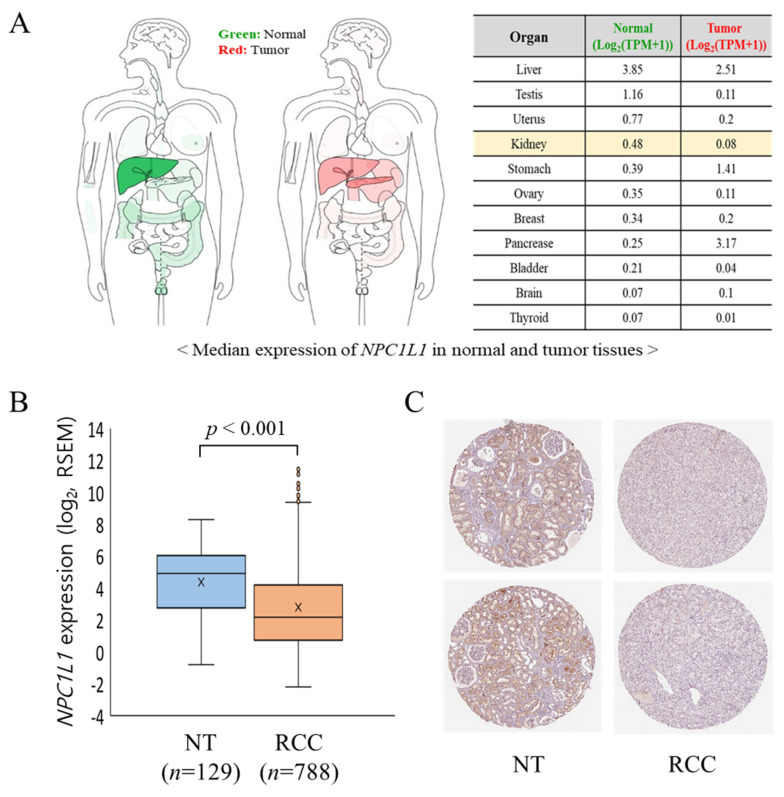
NPC1L1 expression in normal tissues (NT) and RCC tissues. (**A**) Comparison of median *NPC1L1* expression levels between normal (NT) and RCC tissues among various organs using GEPIA2, which presents data as TPM (Transcripts Per Million), showing lower expression in RCC. (**B**) Comparison of mRNA expression levels of *NPC1L1* between normal tissues (orange box: 4.38 ± 2.08) and RCC tissues (sky blue box: 2.81 ± 2.90; *p* < 0.001), indicating significantly lower expression in RCC. RSEM (RNA-Seq by Expectation-Maximization) was used to estimate gene expression levels, providing normalized values for comparison between samples. The data are presented as box-and-whisker plots, with the mean values indicated by an ‘x’. (**C**) Comparison of protein expression levels of NPC1L1 between normal and RCC tissues using data from The Human Protein Atlas, revealing lower expression in RCC. Tissue samples were stained with the CAB070127 antibody. NPC1L1 protein was detected in tubular cells but not in glomerular cells of normal kidney tissue.

The median expression levels of NPC1L1 were compared between normal and tumor tissues across multiple organs using GEPIA2, with a particular focus on the kidney (Figure 1A). In the kidney, *NPC1L1* expression decreased significantly from 0.48 in normal tissue to 0.08 in tumor tissue. This trend of reduced expression in tumor tissues was also observed in other organs, such as the liver and testis, while in contrast, *NPC1L1* expression was higher in tumor tissues of the stomach and pancreas compared to their normal counterparts. When comparing mRNA expression levels, *NPC1L1* expression was significantly higher in normal tissues (4.38 ± 2.08) compared to RCC tissues (2.81 ± 2.90; *p* < 0.001) (Figure 1B). In histological analysis using data from The Human Protein Atlas (Figure 1C), NPC1L1 protein expression was not detected in the glomerular cells of normal kidney tissue but was observed in tubular cells involved in reabsorption. Moreover, consistent with the RNA expression results, NPC1L1 protein expression was lower in RCC tissues than in normal tissues.

**Figure 2:**
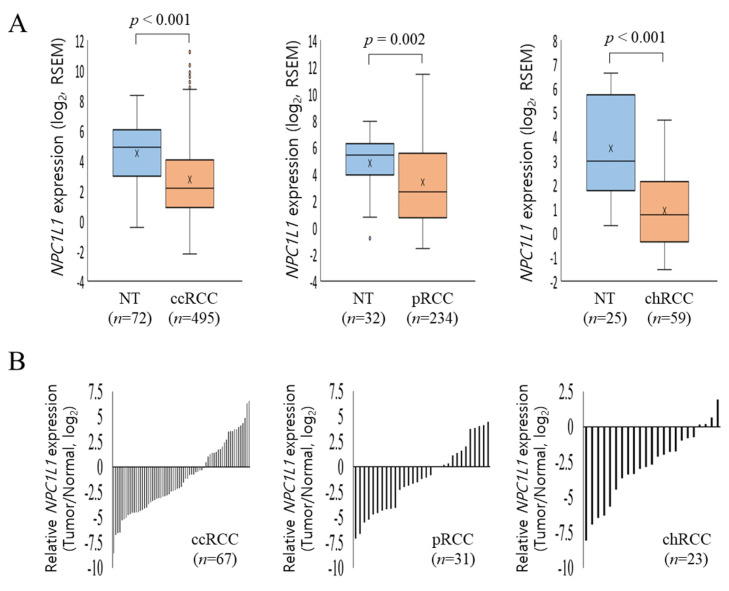
*NPC1L1* expression in RCC subtypes. (**A**) Comparison of *NPC1L1* expression across RCC subtypes. *NPC1L1* expression was lower in tumor tissues (sky blue box) compared to normal tissues (orange box) in all RCC subtypes. In ccRCC, tumor tissues (2.75 ± 2.72) showed lower expression than normal (4.49 ± 1.98; *p* < 0.001). In pRCC, tumor tissues (3.40 ± 3.29) had lower expression than normal (4.80 ± 2.12; *p* = 0.020). In chRCC, tumor tissues (0.93 ± 1.57) also had lower expression than normal (3.51 ± 2.15; *p* < 0.001). The data are presented as box-and-whisker plots, with mean values indicated by an ‘x’. (**B**) Comparison of *NPC1L1* expression between normal and tumor tissues, with tumor tissues normalized to paired non-tumor tissues. In ccRCC, pRCC, and chRCC, *NPC1L1* expression was generally lower in tumor tissues compared to normal tissues, indicating downregulation.

Following the initial analysis of expression between normal and RCC tissues, further investigation was conducted to compare *NPC1L1* expression across RCC subtypes (Figure 2A). In ccRCC, normal tissues showed a significantly higher mean expression of 4.49 ± 1.98 compared to 2.75 ± 2.72 in tumor tissues (*p* < 0.001). Similarly, in pRCC, normal tissues had higher expression levels (4.80 ± 2.12) compared to tumor tissues (3.40 ± 3.29, *p* = 0.020). In chRCC, normal tissues expressed 3.51 ± 2.15 compared to 0.93 ± 1.57 in tumor tissues (*p* < 0.001). To more accurately compare *NPC1L1* expression between normal and tumor tissues, *NPC1L1* expression levels in tumor tissues were normalized to those in paired non-tumor tissues from patients with the same RCC subtype (Figure 2B). In ccRCC and pRCC, the majority of tumor samples showed lower *NPC1L1* expression compared to their corresponding normal tissues, indicating a general downregulation in tumor tissues for these subtypes. Additionally, in chRCC, *NPC1L1* expression was predominantly lower in tumor tissues compared to normal tissues, with most samples showing negative values.

### 3.3. NPC1L1 Expression Based on RCC Stages

**Figure 3:**
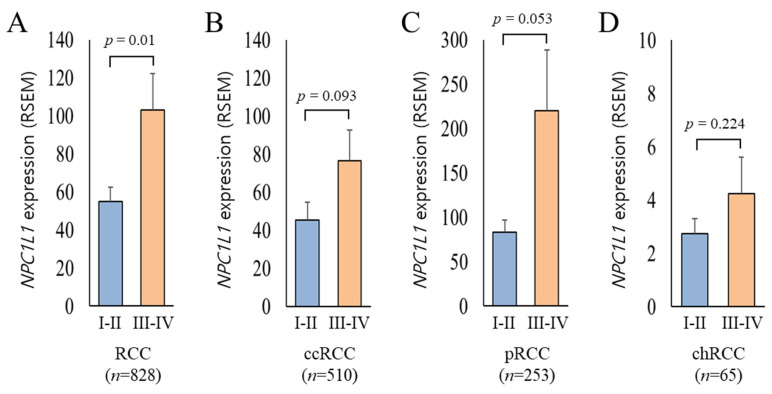
Comparison of *NPC1L1* expression between early and advanced RCC stages. Sky blue boxes represent low stage tumors (I–II) and orange boxes represent high stage tumors (III–IV). (**A**) In the overall RCC group (I–II, *n* = 537; III–IV, *n* = 291), *NPC1L1* expression was significantly higher in advanced stages (mean = 103.16) compared to early stages (mean = 55.05; *p* = 0.005). (**B**–**D**) In ccRCC (I–II, *n* = 303; III–IV, *n* = 207), pRCC (I–II, *n* = 189; III–IV, *n* = 64), and chRCC (I–II, *n* = 45; III–IV, *n* = 20), *NPC1L1* expression showed an upward trend in advanced stages, though the differences were not statistically significant (ccRCC: *p* = 0.093, pRCC: *p* = 0.053, chRCC: *p* = 0.224).

Based on the previous result showing that NPC1L1 expression is generally lower in RCC tissues compared to normal tissues, it was expected that *NPC1L1* expression would decrease further in the high stage (Stage III–IV) relative to the low stage (Stage I–II). However, the analysis showed the opposite results (Figure 3). In the overall RCC group, *NPC1L1* expression was significantly higher in the high stage (mean = 103.16) compared to the low stage (mean = 55.05, *p* = 0.005) (Figure 3A). Additionally, *NPC1L1* expression increased in the high stage across ccRCC, cRCC, and chRCC, showing a trend toward higher expression, though none of the differences were statistically significant (ccRCC: *p* = 0.093, pRCC: *p* = 0.053, chRCC: *p* = 0.224) (Figure 3B–D).

### 3.4. Relationship Between NPC1L1 Expression and Clinical Features

To examine whether the expression level of *NPC1L1* is affected by the age, sex, T, M, N, and pathological stage of RCC patients, the relationship between each clinical feature and the low and high *NPC1L1* expression groups was analyzed (Table 2). The expression of *NPC1L1* was associated with stage T (*p* < 0.001), stage M (*p* < 0.001), stage N (*p* < 0.001), and AJCC stage (*p* < 0.001), indicating that higher *NPC1L1* expression correlated with more advanced metastasis. However, no associations between *NPC1L1* expression and age (*p* = 0.187) and sex (*p* = 0.722) were found.

The association between *NPC1L1* expression and clinical features was further analyzed in different RCC subtypes (Tables S1–S3). In ccRCC, *NPC1L1* expression was significantly associated with M stage (*p* = 0.002) and AJCC stage (*p* < 0.001), with higher expression in advanced stages, but no association was found with age or gender (Table S1). In pRCC, significant associations were observed with gender (*p* < 0.001), T stage (*p* = 0.043), M stage (*p* = 0.010), and N stage (*p* = 0.030), while the association with the AJCC stage was marginal (*p* = 0.066) (Table S2). In chRCC, no significant associations were found between *NPC1L1* expression and clinical characteristics (Table S3).

### 3.5. OS and PFS Based on NPC1L1 Expression in RCC 

**Figure 4:**
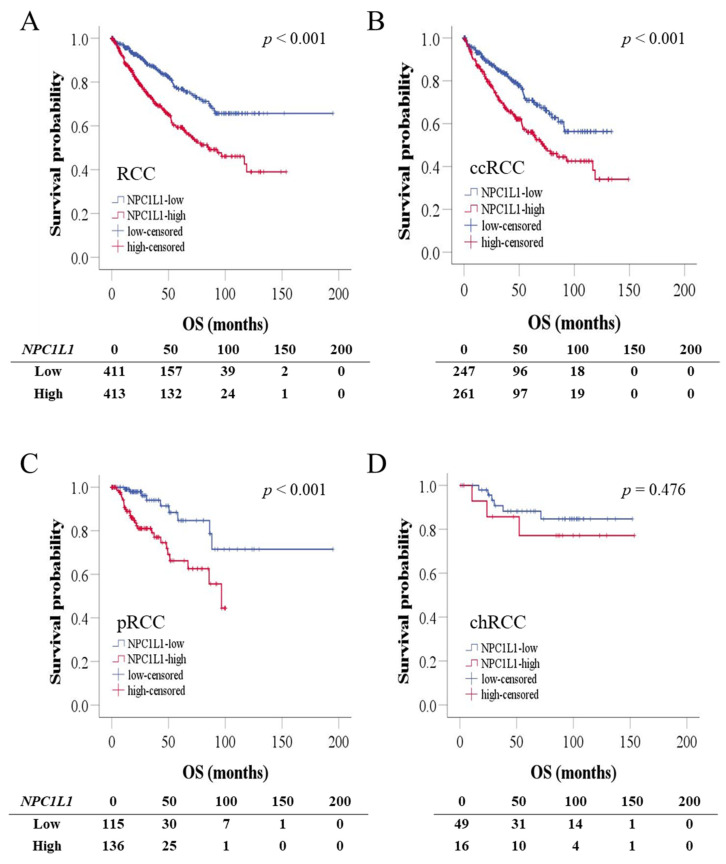
Kaplan–Meier survival analysis of RCC patients based on NPC1L1 expression. The blue line represents the low NPC1L1 expression group, and the red line represents the high NPC1L1 expression group. (**A**) In the overall RCC cohort, high *NPC1L1* expression was associated with shorter survival (89.38 vs. 144.22 months; *p* < 0.001). (**B**–**D**) Similar trends were seen in ccRCC (82.20 vs. 95.78 months; *p* < 0.001) and pRCC (72.99 vs. 157.03 months; *p* < 0.001), but the difference in chRCC was not statistically significant (125.54 vs. 134.52 months; *p* = 0.476).

To determine whether the expression of *NPC1L1* affects the prognosis of RCC patients, Kaplan–Meier survival analysis was used to compare the low and high *NPC1L1* expression groups in relation to OS. OS was assessed in RCC patients based on the median *NPC1L1* expression levels. Patients with high *NPC1L1* expression had shorter survival times compared to those with low expression (Figure 4). In the overall RCC cohort, the mean survival time for the low expression group was 144.22 months compared to 89.38 months for the high expression group (*p* < 0.001) (Figure 4A). For ccRCC, the mean survival time was 95.78 months in the low expression group versus 82.20 months in the high expression group (*p* < 0.001) (Figure 4B). In pRCC, the survival time was 157.03 months in the low expression group compared to 72.99 months in the high expression group (*p* < 0.001) (Figure 4C). However, in chRCC, although a difference was observed between the two groups, with survival times of 134.52 months and 125.54 months, it was not statistically significant (*p* = 0.476) (Figure 4D).

Moreover, to determine how *NPC1L1* influences the progression of the disease, PFS was assessed in RCC patients based on the median *NPC1L1* expression (Figure S2). In the overall RCC cohort, the high expression group showed a significantly shorter mean PFS of 86.25 months compared to the low expression group (*p* < 0.001) (Figure S2A). The same pattern was observed in ccRCC and pRCC, where the high expression group exhibited reduced PFS (both *p* < 0.001) (Figure S2B,C). However, in chRCC, no significant difference in PFS was detected between the expression groups (Figure S2D).

### 3.6. Prognostic Significance of NPC1L1 Expression in RCC Patients

To evaluate the prognostic significance of *NPC1L1* expression in RCC patients, univariable and multivariable Cox proportional hazards models were conducted (Table 3). In the overall cohort, high *NPC1L1* expression was a significant prognostic factor for OS, with worse survival outcomes compared to low expression in both the univariable (HR = 2.115, *p* < 0.001) and multivariable analyses (HR = 1.664, *p* < 0.001). In ccRCC, high *NPC1L1* expression was associated with poor survival in the univariable analysis (HR = 1.771, *p* < 0.001), though the result was not statistically significant in the multivariable analysis (HR = 1.315, *p* = 0.098). In pRCC, high *NPC1L1* expression was significantly associated with worse survival in both the univariable (HR = 3.446, *p* = 0.001) and multivariable analyses (HR = 3.446, *p* = 0.001). However, in chRCC, no significant association was found between *NPC1L1* expression and survival in either the univariable (HR = 1.647, *p* = 0.481) or multivariable analysis (HR = 3.058, *p* = 0.143).

### 3.7. OS According to Combined NPC1L1 Expression and Stage in RCC

**Figure 5:**
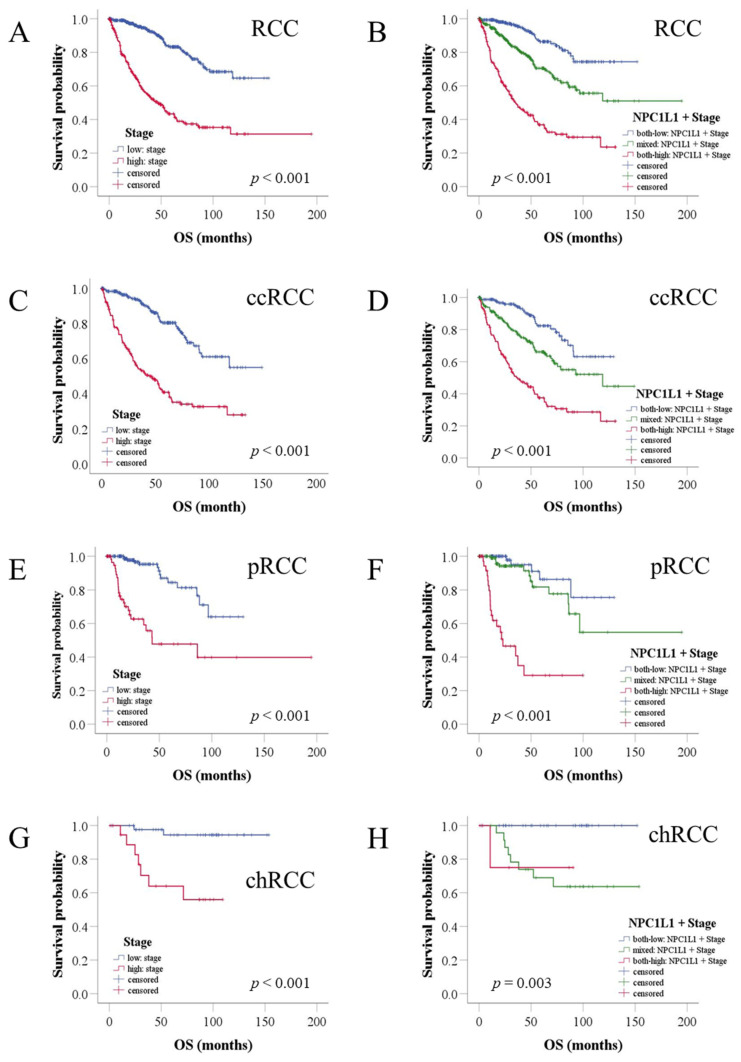
Kaplan–Meier analysis of OS in RCC patients based on combined NPC1L1 expression and tumor stage. The blue line represents the group with low NPC1L1 expression and low stage, the red line represents the group with high NPC1L1 expression and high stage, and the green line represents the mixed group. (**A**,**B**) High-stage patients had a median survival of 47.08 months, but adding NPC1L1 expression reduced this to 35.18 months. (**C**–**H**) In ccRCC, survival decreased from 43.96 to 37.25 months, in pRCC from 43.2 to 22.92 months, and in chRCC, the 75th percentile survival dropped from 30.25 to 10.69 months in the high NPC1L1/high-stage group.

To assess whether *NPC1L1* expression, combined with stage, could better identify patients with poorer prognoses, the patients were divided into three groups based on *NPC1L1* expression and stage (Figure 5, Table S4). Patients with low *NPC1L1* and low stage, mixed *NPC1L1* expression and stage levels, and high *NPC1L1* and high-stage were compared. In the overall RCC cohort, high-stage patients had a median survival of 47.08 months (Figure 5A). However, when *NPC1L1* expression was considered, the high *NPC1L1*/high-stage group had a shorter median survival of 35.18 months (Figure 5B), indicating that adding *NPC1L1* expression helps identify patients with a worse prognosis within the high-stage group.

For RCC subtypes, high-stage ccRCC patients had a median survival of 43.96 months, while the high *NPC1L1*/high-stage group had a shorter survival of 37.25 months (Figure 5C,D). In pRCC, high-stage patients had a median survival of 43.2 months, while the high *NPC1L1*/high-stage group had a much shorter survival of 22.92 months (Figure 5E,F). In chRCC, where the median could not be calculated due to low mortality, the 75th percentile was used. High-stage chRCC patients had a survival of 30.25 months, while the high *NPC1L1*/high stage group had 10.69 months (Figure 5G,H). Throughout all subtypes, *NPC1L1* expression combined with stage helped better identify patients with poor outcomes compared to using stage alone.

### 3.8. Gene Effect Scores for NPC1L1 in Renal Cancer Cell Lines

**Figure 6:**
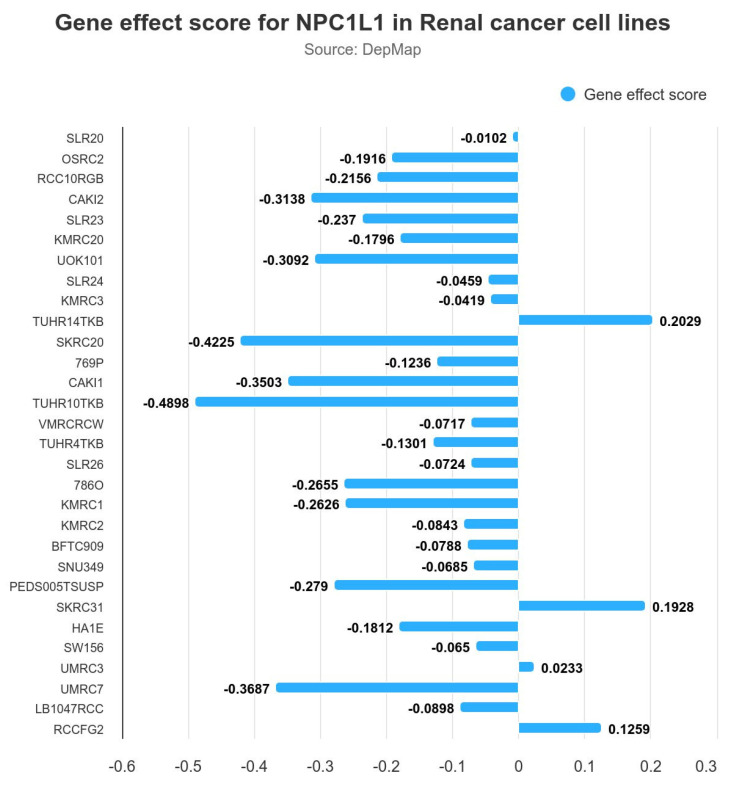
Gene effect scores for NPC1L1 in various renal cancer cell lines. The negative gene effect scores indicate that NPC1L1 is essential for the survival of several cell lines, with the lowest score being −0.4898 in RCC10RGB. A few cell lines showed minimal or no dependency on NPC1L1, with positive gene effect scores such as 0.2029 in BFTC909.

To determine whether NPC1L1 is a crucial molecule in the progression of RCC, gene effect scores for NPC1L1 were assessed across several renal cancer cell lines using data from DepMap, provided by Ualcan (Figure 6). A negative gene effect score indicates that the cell line is highly dependent on the gene for survival, as depletion of the gene reduces cell viability. Conversely, a positive score reflects minimal dependency on the gene, with little impact on survival upon depletion. The majority of cell lines, including OSRC2, RCC10RGB, and CAKI2, exhibited negative gene effect scores, with the lowest score being approximately −0.4898, indicating that NPC1L1 may be essential for the survival of these cancer cells. In contrast, a few cell lines showed minimal or no dependency on NPC1L1, as reflected by slightly positive gene effect scores, such as 0.2029 and 0.1928. 

## 4. Discussion

NPC1L1 plays a crucial role in cholesterol absorption in the intestines and is targeted by ezetimibe, a medication commonly prescribed for managing dyslipidemia that does not respond well to statins. Despite its known function in lipid metabolism, the expression patterns and prognostic significance of NPC1L1 in RCC and other cancers remain largely unexplored. 

The expression of *NPC1L1* is known to vary, with lower levels reported in hepatocellular carcinoma (HCC) but higher levels in pancreatic and colon cancers [15,16,17]. In the present study, similar to HCC, significant downregulation of *NPC1L1* RNA expression is observed in RCC overall, as well as in the subtypes ccRCC, pRCC, and chRCC, with tumor tissues consistently showing lower expression levels compared to normal tissues (Figure 1A,B and Figure 2A,B). Immunohistochemistry analysis further confirmed that NPC1L1 expression was lower in RCC tissues compared to normal tissues, aligning with the RNA expression results (Figure 1C). These findings suggest that NPC1L1 downregulation is a common feature of RCC, potentially reflecting metabolic changes associated with RCC development. The Warburg effect, which involves a shift from oxidative phosphorylation to glycolysis for energy production, plays a crucial role in metabolic reprogramming [18]. This reprogramming leads to the accumulation of cholesterol and lipid droplets, primarily through the increased use of the Very-Low-Density Lipoprotein Receptor (VLDL-R) and Scavenger Receptor Class B Member 1 (SR-B1), which support the rapid proliferation of RCC [19,20]. Thus, the reduced expression of *NPC1L1* may reflect its limited role in the development of RCC, potentially allowing the activation of alternative metabolic pathways critical for cancer development.

Few studies have explored the relationship between NPC1L1 expression and clinical features related to disease progression. For example, in colon cancer, *NPC1L1* expression is significantly associated with advanced stages, particularly in patients with lymph node involvement and higher AJCC stage, suggesting its role in cancer progression [15]. In contrast, in hepatocellular carcinoma (HCC), low NPC1L1 expression correlates with a higher incidence of poorly differentiated tumors, vascular invasion, and an increased number of patients with advanced pathological stages [17]. In RCC, although *NPC1L1* levels were expected to be lower in advanced stages due to its reduced expression in tumor tissues compared to normal tissues, it was unexpectedly found to increase in higher stages (Figure 3A,D). This trend was observed across all RCC subtypes, though none reached individual statistical significance, with pRCC being marginally significant. Moreover, higher *NPC1L1* expression in RCC was closely associated with aggressive disease characteristics, showing significant correlations across advanced T, M, N stages and AJCC stage (Table 2). Subtype analysis also revealed strong associations in ccRCC and pRCC, particularly related to metastasis and AJCC stage (Tables S1–S3). The increased *NPC1L1* expression in RCC, particularly associated with higher M stages, suggests a role in facilitating metastatic processes. This upregulation may facilitate the fulfillment of the increased cholesterol demands of metastatic cancer cells, supplementing alternative pathways like SR-B1 and VLDL-R. Therefore, as the tumor spreads, NPC1L1 may support the metabolic flexibility needed for enhanced tumor aggressiveness and survival. Taken together, these findings suggest that the function of NPC1L1 is cancer type-specific, assuming different roles in various cancers such as RCC, colon cancer, and HCC. Specifically, *NPC1L1* downregulation may reflect a metabolic adaptation in RCC development, where the Warburg effect shifts energy production towards glycolysis, and the effective capabilities of SR-B1 and VLDL-R to transfer cholesterol in bulk from HDL and VLDL—unlike the absorption of individual cholesterol molecules by NPC1L1—adequately meets the tumor’s initial cholesterol demands. As RCC progresses to later stages, increased cholesterol requirements may lead to *NPC1L1* upregulation, supporting tumor progression and metastasis. Similarly, snail family transcriptional repressor 2 (SNAI2), an epithelial-mesenchymal transition (EMT) related transcription factor, though downregulated overall in RCC, is associated with poorer outcomes when expressed at high levels [21]. Further studies are needed to confirm these insights in the context of metabolic and molecular adaptations in RCC development and progression.

Several prognostic markers have been proposed for RCC. The overexpression of Ki67 and HIF1A in clear cell RCC has been associated with poor survival, and IMP3-positive clear cell RCC has been found to carry a higher risk of metastasis compared to IMP3-negative clear cell RCC [22,23,24]. Additionally, the overexpression of FASN, which is involved in fatty acid synthesis, has been correlated with poor prognosis in RCC [25]. These studies show that identifying various prognostic markers is crucial for evaluating RCC progression. In the present study, *NPC1L1* was identified as a potential prognostic marker in RCC, particularly in ccRCC and pRCC, where high expression levels were associated with significantly worse OS and PFS (Figure 4A–C and Figure S2A–C, Table 3). In the case of chRCC, the lack of statistical significance may be due to the smaller sample size, potentially limiting the power of the analysis (Figure 4D and Figure S2D, Table 3). Moreover, the combination of *NPC1L1* expression with RCC stage provided a more precise prognostic indicator, especially for high-stage RCC. Patients with both high *NPC1L1* expression and advanced stage had significantly shorter survival times than those with advanced stage alone (Figure 5A,B). This pattern was consistent across RCC subtypes, showing that combining *NPC1L1* expression with tumor stage helps to better identify high-risk patients (Figure 5C–H). To confirm the potential of NPC1L1 as a prognostic marker in RCC, gene effect scores in several renal cancer cell lines were analyzed. Negative scores observed in cell lines, such as OSRC2, RCC10RGB, and CAKI2, suggest that NPC1L1 is essential for the survival of these cancer cells (Figure 6). This suggests that NPC1L1 plays an important role in supporting tumor metabolism and growth, further validating its significance in RCC progression. Therefore, NPC1L1 is an independent marker for evaluating the prognosis of patients with RCC. Moreover, given the role of NPC1L1 as the target of ezetimibe, exploring the use of ezetimibe as an adjuvant therapy in RCC could be one potential approach to inhibiting tumor progression and metastasis.

## 5. Strengths and Limitations

This study provides a comprehensive analysis of NPC1L1 as a prognostic marker in RCC, based on a large cohort of 828 patients across multiple subtypes. Moreover, the integration of bioinformatic data from GEPIA2, The Human Protein Atlas, and UALCAN further strengthens the analysis. These analyses helped us better understand the potential involvement of NPC1L1 in the development and progression of RCC. Finally, the analysis of the combination of NPC1L1 expression and tumor stage demonstrated that although tumor stage is a critical prognostic indicator, high NPC1L1 expression also significantly influences patient outcomes, providing additional prognostic value beyond traditional staging factors. This provides a foundation for further research on NPC1L1 as a therapeutic target in RCC, with the potential to enhance treatment outcomes. Despite its strengths, this study has several limitations. First, RNA expression levels in the present study could not be verified through protein levels, as these may not always correlate directly. To examine the relationship between RNA and protein expression in the kidney, we analyzed data for NPC1L1 from the consensus dataset, The Human Protein Atlas (HPA), and protein expression dataset, which rank expression levels across tissues in descending order (Figure S3). The datasets show high NPC1L1 expression levels in tissues like the duodenum and small intestine, with consistent RNA and protein levels. In the kidney, both RNA and protein levels of NPC1L1 are low, suggesting a proportional relationship. In contrast, in the testis, low RNA levels do not correspond with detectable proteins, demonstrating that low mRNA expression does not always result in protein expression. This observation supports the possibility of a proportional relationship between mRNA and protein levels for NPC1L1 in RCC. Second, this study lacks direct experimental validation of the functional role of NPC1L1 in the development and progression of RCC through surgically resected tissues and cultured cell line experiments. Although we assessed gene effect scores across several renal cancer cell lines, which indicated that NPC1L1 knockout significantly reduced cell survival, further validation using direct in vitro and in vivo studies is still needed. These findings will establish a basis for future studies to investigate the molecular mechanisms of NPC1L1 relevant to the development and progression of RCC.

## 6. Conclusions

This study demonstrates for the first time that high *NPC1L1* expression in RCC is associated with poor survival, establishing *NPC1L1* as an independent prognostic marker. These findings suggest that clinicians could use *NPC1L1* expression to identify RCC patients with a poor prognosis. Additionally, the study shows that *NPC1L1* is upregulated in high-stage RCC compared to low-stage RCC, providing insights into how RCC progression may involve cholesterol metabolism and emphasizing the need for further investigation into the role of NPC1L1 in RCC progression.

## Figures and Tables

**Table 1:** General characteristics of patients with RCC.

Histological Type (*n* = 828)		Clear Cell	Papillary	Chromophobe	Total (%)
		510 (61.6)	253 (30.6)	65 (7.8)	
Overall survival months (mean ± SD)		44.25 ± 32.54	33.38 ± 29.74	70.27 ± 40.05	42.97 ± 33.66
Age (mean ± SD)		60.49 ± 12.05	61.64 ± 12.13	51.15 ± 14.10	60.11 ± 12.52
Sex	Male	325 (63.7)	190 (75.1)	38 (58.5)	553 (66.8)
	Female	185 (36.3)	63 (24.9)	27 (41.5)	275 (33.2)
T stage	T1–T2	320 (62.7)	195 (77.1)	45 (69.2)	560 (67.6)
	T3–T4	190 (37.3)	56 (22.1)	20 (30.8)	266 (32.1)
	Unknown	0 (0.0)	2 (0.8)	0 (0.0)	2 (0.3)
M stage	M0	401 (78.6)	83 (32.8)	34 (52.3)	518 (62.6)
	M1	78 (15.3)	9 (3.6)	2 (3.1)	89 (10.7)
	Unknown	31 (6.1)	161 (63.6)	29 (44.6)	221 (26.7)
N stage	N0	228 (44.7)	46 (18.2)	39 (60.0)	313 (37.8)
	N1–N2	16 (3.1)	27 (10.7)	5 (7.7)	48 (5.8)
	Unknown	266 (52.2)	180 (71.1)	21 (32.3)	467 (56.4)
AJCC stage	Stage I	249 (48.8)	168 (66.4)	20 (30.8)	437 (52.8)
	Stage II	54 (10.6)	21 (8.3)	25 (38.5)	100 (12.0)
	Stage III	124 (24.3)	49 (19.4)	14 (21.5)	187 (22.6)
	Stage IV	83 (16.3)	15 (5.9)	6 (9.2)	104 (12.6)

AJCC, American Joint Committee on Cancer; RCC, renal cell carcinoma.

**Table 2:** Association between NPC1L1 expression and clinical characteristics of RCC patients.

Characteristics	*n*	*NPC1L1* (Low)	*NPC1L1* (High)	*p*-Value
Age (years)	828			*p* = 0.187
<60		219	200	
≥60		195	214	
Sex	828			*p* = 0.712
Male		279	274	
Female		135	140	
T stage	826			*p* < 0.001
T1-T2		308	252	
T3-T4		105	161	
M stage	607			*p* < 0.001
M0		266	252	
M1		25	64	
N stage	361			*p* < 0.001
N0		160	153	
N1-N2		13	35	
AJCC stage	828			*p* < 0.001
Stage I–II		301	236	
Stage III–IV		113	178	

AJCC, American Joint Committee on Cancer; RCC, renal cell carcinoma.

**Table 3:** Univariable and multivariable analysis of prognostic factors in RCC patients for OS.

RCC (n = 828)	Univariable		Multivariable	
	Hazard Ratio (95% CI)	*p*-Value *	Hazard Ratio (95% CI)	*p*-Value *
Age < 60 (vs. ≥ 60)	1.774 (1.348–2.335)	*p* < 0.001	1.519 (1.151–2.004)	*p* = 0.003
Sex Male (vs. Female)	1.091 (0.825–1.443)	*p* = 0.541	1.145 (0.864–1.516)	*p* = 0.347
Stage I + II (vs. III + IV)	4.853 (3.640–6.470)	*p* < 0.001	4.385 (3.278–5.867)	*p* < 0.001
NPC1L1 Low (vs. High)	2.115 (1.598–2.801)	*p* < 0.001	1.664 (1.252–2.211)	*p* < 0.001
**ccRCC** **(n = 510)**	**Univariable**		**Multivariable**	
	**Hazard Ratio** **(95% CI)**	*p*-value *	**Hazard Ratio** **(95% CI)**	*p*-value *
Age < 60 (vs. ≥ 60)	1.833 (1.341–2.505)	*p* < 0.001	1.573 (1.144–2.164)	*p* = 0.005
Sex Male (vs. Female)	1.043 (0.763–1.426)	*p* = 0.792	1.059 (0.770–1.455)	*p* = 0.725
Stage I + II (vs. III + IV)	4.010 (3.640–6.470)	*p* < 0.001	3.634 (2.598–5.083)	*p* < 0.001
NPC1L1 Low (vs. High)	1.771 (2.890–5.564)	*p* < 0.001	1.315 (0.951–1.810)	*p* = 0.098
**pRCC** **(n = 253)**	**Univariable**		**Multivariable**	
	**Hazard Ratio** **(95% CI)**	*p*-value *	**Hazard Ratio** **(95% CI)**	*p*-value *
Age < 60 (vs. ≥ 60)	1.087 (0.566–2.085)	*p* = 0.803	1.035 (0.534–2.005)	*p* = 0.919
Sex Male (vs. Female)	1.594 (0.765–3.321)	*p* = 0.213	1.239 (0.592–2.596)	*p* = 0.569
Stage I + II (vs. III + IV)	6.268 (3.240–12.128)	*p* < 0.001	6.309 (3.258–12.217)	*p* < 0.001
NPC1L1 Low (vs. High)	3.446 (1.626–7.288)	*p* = 0.001	3.446 (1.606–7.397)	*p* = 0.001
**chRCC** **(n = 65)**	**Univariable**		**Multivariable**	
	**Hazard Ratio** **(95% CI)**	*p*-value *	**Hazard Ratio** **(95% CI)**	*p*-value *
Age < 60 (vs. ≥ 60)	2.284 (0.612–8.526)	*p* = 0.219	3.194 (0.635–16.066)	*p* = 0.159
Sex Male (vs. Female)	0.643 (0.160–2.575)	*p* = 0.532	2.392 (0.395–14.476)	*p* = 0.342
Stage I + II (vs. III + IV)	10.223 (2.120–49.293)	*p* =0.004	14.727 (2.241–96.773)	*p* = 0.006
NPC1L1 Low (vs. High)	1.647 (0.412–6.593)	*p* = 0.481	3.058 (0.654–18.810)	*p* = 0.143

* *p*-values were derived from the Cox proportional hazard model; RCC, renal cell carcinoma; ccRCC, clear cell renal cell carcinoma; pRCC, papillary renal cell carcinoma; chRCC, chromophobe renal cell carcinoma.

## Data Availability

The data used in this study were obtained from cBioPortal (https://www.cbioportal.org/), which provides access to The Cancer Genome Atlas (TCGA) datasets.

## Supplementary Materials

The following supporting information can be downloaded at: https://www.mdpi.com/article/10.3390/life14111444/s1, Figure S1: Data collection flowchart for RCC patient cohort; Figure S2: Kaplan-Meier analysis of progression-free survival (PFS) in RCC patients based on *NPC1L1* expression; Figure S3: NPC1L1 expression across various human tissues; Table S1: Association between NPC1L1 expression and clinical characteristics of ccRCC patients; Table S2: Association between NPC1L1 expression and clinical characteristics of pRCC patients; Table S3: Association between NPC1L1 expression and clinical characteristics of chRCC patients; Table S4: Number at risk for Kaplan-Meier analysis of overall survival in RCC patients by combined NPC1L1 expression and tumor stage.

## Abbreviations

AJCC	American Joint Committee on Cancer
HCC	Hepatocellular Carcinoma
HPA	The Human Protein Atlas
ccRCC	Clear Cell Renal Cell Carcinoma
chRCC	Chromophobe Renal Cell Carcinoma
NPC1L1	Niemann-Pick C1-Like 1
OS	Overall Survival
PFS	Progression-Free Survival
pRCC	Papillary Renal Cell Carcinoma
RCC	Renal Cell Carcinoma
SR-B1	Scavenger Receptor Class B Member 1
TCGA	The Cancer Genome Atlas
VLDL-R	Very-Low-Density Lipoprotein Receptor
