# Supplementary material for: Niemann–Pick C1-like 1 as a Prognostic Marker in Renal Cell Carcinoma: A Retrospective Cohort Study
Source: Life (Basel). 2024 Nov 7;14(11):1444. doi: 10.3390/life14111444 (PMC11595514; doi:10.3390/life14111444)
Supplement: Supplementary file 1 [file life-14-01444-s001.zip › life-3260987-supplementary.pdf]

Figure S1. Data collection flowchart for RCC patient cohort

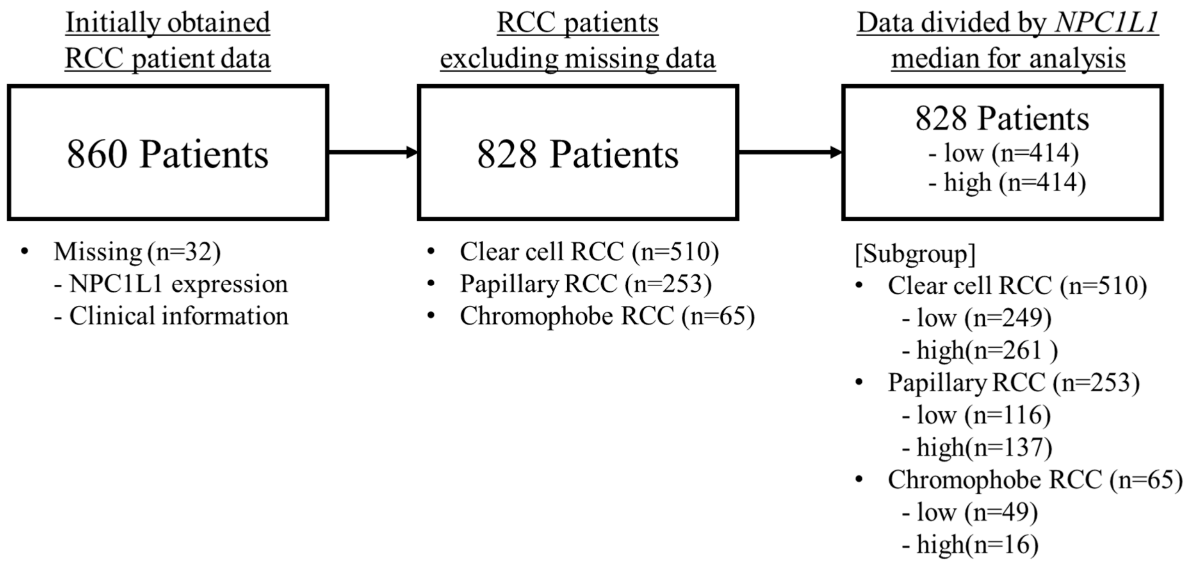

Figure S2. Kaplan-Meier analysis of progression-free survival (PFS) in RCC patients based on *NPC1L1* expression. (A) In the overall RCC cohort, high *NPC1L1* expression was associated with shorter progression-free survival (mean time: 86.247 vs. 144.974 months; $p < 0.001$). (B-D) Similar trends were seen in ccRCC (mean time: 70.967 vs. 99.793 months; $p < 0.001$) and pRCC (mean time: 71.783 vs. 138.420 months; $p < 0.001$), but the difference in chRCC was not statistically significant (mean time: 133.238 vs. 113.932 months; $p = 0.636$).

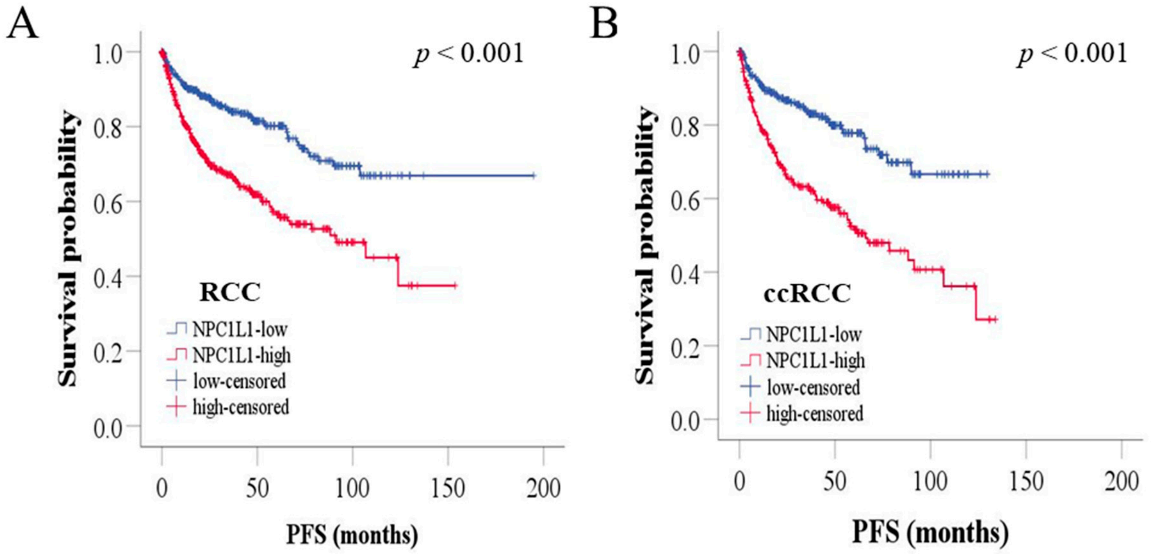

NPC1L1	0	50	100	150	200	0	50	100	150	200
**Low**	**409**	**140**	**34**	**1**	**0**	**246**	**84**	**14**	**0**	**0**
**High**	**411**	**104**	**17**	**1**	**0**	**260**	**76**	**12**	**0**	**0**

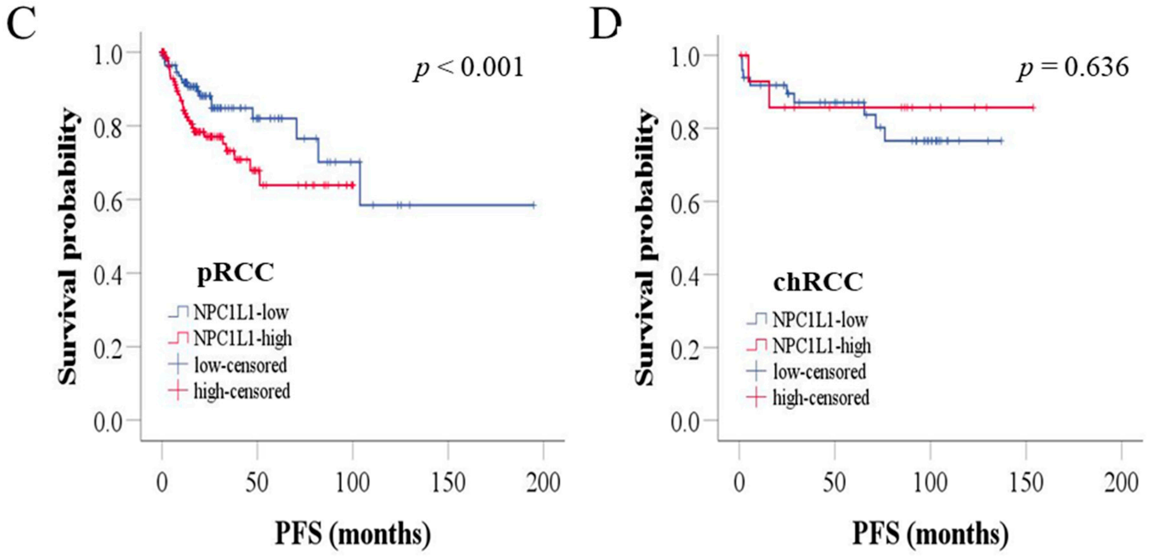

NPC1L1	0	50	100	150	200	0	50	100	150	200
**Low**	**114**	**25**	**7**	**1**	**0**	**49**	**31**	**13**	**0**	**0**
**High**	**135**	**19**	**1**	**0**	**0**	**16**	**9**	**4**	**1**	**0**

Figure S3. NPC1L1 expression across various human tissues. The left panel represents RNA expression levels from the consensus dataset, showing high expression in the duodenum and small intestine, moderate levels in the liver, and lower or minimal levels in other tissues, measured in normalized transcripts per million (nTPM) (https://www.proteinatlas.org/) (accessed on 24 October 2024). The consensus dataset, combining the Human Protein Atlas (HPA) and Genotype-Tissue Expression (GTEx) transcriptomics data, presents normalized RNA expression levels (nTPM) across 55 tissue types, grouped by common functional features. The middle panel illustrates RNA expression from the HPA dataset, confirming similar high expression in the duodenum and small intestine and low expression in tissues such as the kidney and testis. The right panel displays protein expression detected through immunohistochemistry (IHC), indicating a high protein level in the duodenum and small intestine, moderate levels in the liver and appendix, and low or undetectable levels in tissues like the kidney and cerebral cortex.

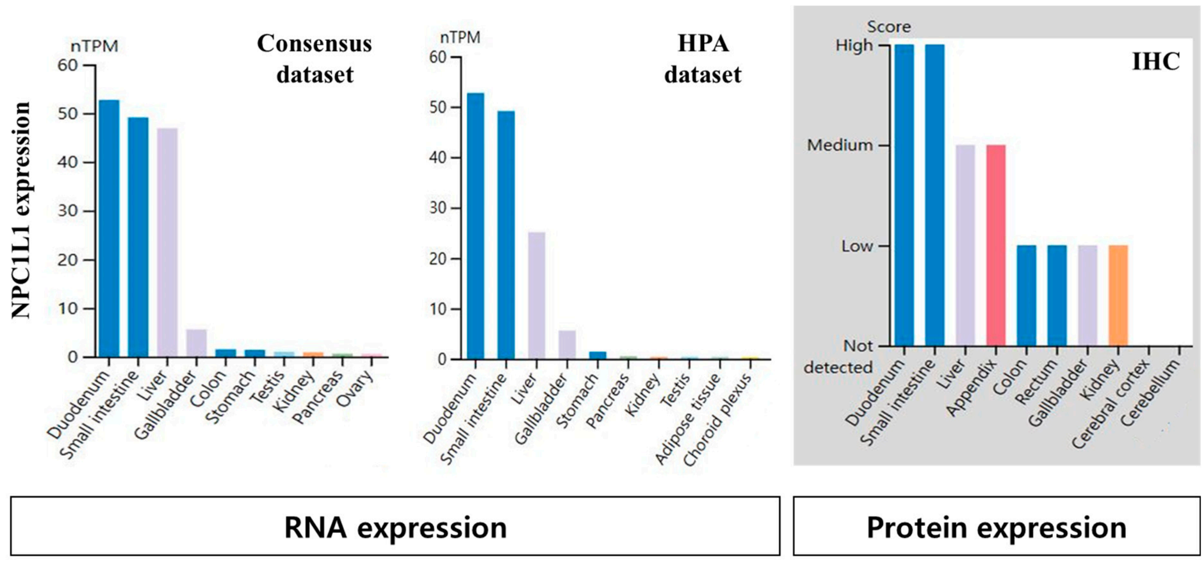

Table S1. Association between *NPC1L1* expression and clinical characteristics of ccRCC patients

Characteristics	N	Low	High	*p*-value
Age (years)	510			$p = 0.375$
<60		130	126	
≥60		119	135	
Sex	510			$p = 0.389$
Male		154	171	
Female		95	90	
T stage	510			$p = 0.187$
T1-T2		197	128	
T3-T4		123	62	
M stage	479			$p = 0.002$
M0		202	199	
M1		24	54	
N stage	244			$p = 0.077$
N0		109	119	
N1-N2		4	12	
AJCC stage	510			$p < 0.001$
Stage I-II		173	130	
Stage III-IV		76	131	

AJCC, American Joint Committee on Cancer; ccRCC, clear cell RCC

Table S2. Association between *NPC1L1* expression and clinical characteristics of pRCC patients

Characteristics	N	Low	High	*p*-value
Age (years)				*p* = 0.747
<60	253	54	61	
≥60		62	76	
Sex				*p* < 0.001
Male	253	99	91	
Female		17	46	
T stage				*p* = 0.043
T1-T2	251	96	99	
T3-T4		19	37	
M stage				*p* = 0.010
M0	92	37	46	
M1		0	9	
N stage				*p* = 0.030
N0	73	20	26	
N1-N2		5	22	
AJCC stage				*p* = 0.066
Stage I-II	253	93	96	
Stage III-IV		23	41	

AJCC, American Joint Committee on Cancer; pRCC, papillary RCC

Table S3. Association between *NPC1L1* expression and clinical characteristics of chRCC patients

Characteristics	N	Low	High	*p*-value
Age (years)	65			*p* = 0.438
<60		35	13	
≥60		14	3	
Sex	65			*p* = 0.122
Male		26	12	
Female		23	4	
T stage	65			*p* = 0.502
T1-T2		35	10	
T3-T4		14	6	
M stage	36			*p* = 0.331
M0		27	7	
M1		1	1	
N stage	44			*p* = 0.979
N0		31	8	
N1-N2		4	1	
AJCC stage	65			*p* = 0.502
Stage I-II		35	10	
Stage III-IV		14	6	

AJCC, American Joint Committee on Cancer; chRCC, chromophobe RCC

Table S4. Number at risk for Kaplan-Meier analysis of overall survival in RCC patients by combined *NPC1L1* expression and tumor stage

Number at risk										
RCC (months)										
Stage	0	50	100	150	200	Stage + *NPC1L1*	0	50	100	150	200
Low	526	200	45	2	0	Low	293	118	31	1	0
High	287	82	16	1	0	Mixed	344	118	21	2	0
						High	176	46	9	0	0
ccRCC (months)											
Stage	0	50	100	150	200	Stage + *NPC1L1*	0	50	100	150	200
Low	301	129	24	0	0	Low	171	72	14	0	0
High	207	63	13	0	0	Mixed	206	80	14	0	0
						High	131	40	9	0	0
pRCC (months)											
Stage	0	50	100	150	200	Stage + *NPC1L1*	0	50	100	150	200
Low	188	44	6	0	0	Low	92	24	5	0	0
High	63	11	2	1	0	Mixed	119	26	3	1	0
						High	40	5	0	0	0
chRCC (months)											
Stage	0	50	100	150	200	Stage + *NPC1L1*	0	50	100	150	200
Low	45	32	16	2	0	Low	35	24	12	1	0
High	20	9	2	0	0	Mixed	24	15	6	1	0
						High	6	2	0	0	0

ccRCC, clear cell RCC; pRCC, papillary RCC; chRCC, chromophobe RCC
